# Research on the interaction between trench material and pipeline under fault displacement

**DOI:** 10.1038/s41598-024-57936-9

**Published:** 2024-05-30

**Authors:** Ming Yang, Dongyuan Wang, Haidong Jia, Wenjun Hu, Yu Zhao, Jungfeng Tang

**Affiliations:** 1 China Petroleum West Pipeline Co., Ltd., Urumqi, Xinjiang China; 2 China National Petroleum Pipeline No. 1 Engineering Company, Langfang, Hebei China; 3 China Petroleum Engineering & Construction Southwest Company, Chengdu, Sichuan China; 4 Institute of Mountain Hazards and Environment, Chinese Academy of Science, Chengdu, Sichuan China; 5 College of Civil Engineering, Sichuan Agricultural University, Dujiangyan, Sichuan China

**Keywords:** Pipe–soil interaction, Foam concrete, Buried pipeline, Normal fault, Protective effect, Civil engineering, Energy infrastructure

## Abstract

With the large-scale construction of oil and gas pipelines, the safety issues of long-distance buried pipelines in the service and construction have become increasingly prominent. The complex geological and topographical conditions of the special zone will put forwards extremely high requirements on pipe trench laying backfill materials and construction technology. For example, pipelines are inevitable to cross the active fault, while the trench backfilled with soil has limitations in protecting them from failure under the active fault displacement caused by the earthquake. Therefore, it is necessary to study the pipe–soil interaction mechanism, determine the stress state of the pipeline and propose a new backfilling material that can protect the pipeline from failure. Foam concrete (FC) provides a new choice to backfill the buried pipeline trench due to its high-homogeneity, lightweight, controllable-strength, and self-compacting. To further determine the applicability of the FC, the pipe-FC interaction mechanism is studied. Then, a FE model of the FC-pipeline-soil interaction system is established by Abaqus to quantitatively analyze the applicability of the FC based on the experimental data of the mechanical performance of the FC. It proves that using FC as trench backfill material has a noticeable protective effect on the pipeline under the earthquake-induced displacement of the normal fault. Furthermore, FC has a better protective effect on the pipeline subjected to compressive than tensile. Therefore, the reference for applying FC in trench backfilling of pipelines crossing normal fault is provided.

## Introduction

The buried pipelines are usually used for long-distance transportation of oil and natural gas resources due to their convenience and safety. Nevertheless, with the large-scale construction of buried pipelines, pipelines will inevitably cross the active fault, and the earthquake-induced fault displacement poses a considerable threat to the safe operation of pipelines when the trench is backfilled with soil^1–7^. It has been recognized that the complex pipe–soil interaction near the fault region may strongly influence pipeline response.

Many researchers have carried out a series of studies on the mechanism of the pipe–soil interaction under the fault displacement using experimental, theoretical and numerical methods. Karamitros et al.^8^ presented an analytical methodology, using a combination of beam-on-elastic-foundation and beam theory, to compute the pipeline axial force, bending moment and maximum strain. Daiyan et al.^9^ simulated the soil around a non-deformable pipeline with elastic–plastic 3D solid finite elements to investigate the soil-pipe interaction and load transfer mechanisms. Jalali and Rofooei^10^ studied the pipe–soil interaction of buried pipelines across faults by designing scale test and numerical simulation methods, obtained the deformation law of pipeline and soil under fault dislocation, and determined the maximum equivalent soil pipe interaction force and its distribution along the pipeline. Vazouras et al.^11^.investigated the pipe–soil interaction and the performance of pipelines under the strike-slip movement and developed a closed- form mathematical solution for the force–displacement relationship of a buried pipeline subjected to tension for pipelines of finite and infinite lengths. Dong et al.^12^. developed a coupled large deformation finite element (LDFE) framework and discussed the effects of the interaction rate and hence drainage condition on the p-y curve and proposed an empirical relationship between the ultimate resistance and the normalized velocity of the pipeline. Cheng et al.^13,14^ analyzed the pipe–soil interaction and discussed the pipeline failure modes and the protective effect of the soil by established a 3D FE model. Saif et al.^15–18^ studied the influence of using different low stiffness materials, such as the Tire-Derived Aggregate (TDA), as trench fillings for buried pipes under different loads and conditions. These studies suggest that TDA can be an effective material for reducing stress and strain on buried concrete pipes, particularly in areas with seismic activity. The interaction between the pipeline and the soil can be significantly influenced by seismic activity. Güllü et al.^19,20^ used deterministic approaches and the total probability theorem to predict ground motion and emphasized prediction equations can be crucial in predicting how seismic events might affect this interaction.

Although much research on the pipe–soil interaction has been carried out under fault displacement, there are few studies on the phenomenon of common contact deformation or contact separation between the pipeline and soil under fault displacement due to the discreteness of soil. Additionally, the pipeline crossing area is complex, and we need to pay more attention to the following problems:

(1) Under the fault displacement, the pipeline and soil will separate due to the great difference of deformation stiffness between the pipeline and soil. As the increase of the fault displacement, the pipe–soil interaction will transform from coordinated to non-coordinated deformation, the stress and strain of the pipeline will increase obviously, which may cause the pipeline failure under the fault displacement.

(2) The traditional pipe trench is generally backfilled with fine sand and soil (hereinafter collectively referred to as soil), which maximum particle size is less than 20mm^21,22^. However, the geological and topography conditions are complicated in some zones, which results in a lack of soil in construction sites. In addition, if the trench space is too narrow for the soil to be fully compacted, which can lead to land subsidence and collapse and cause pipeline failure^1^. Meanwhile, soil inhomogeneity is easy to produce stress concentration on the pipeline, which poses a potential hazard to pipeline safety.

In view of the above problems, the pipe–soil interaction under fault displacement is studied in detail. The pipe–soil interaction process is divided into coordinated and non-coordinated deformation. Based on the elastic foundation beam theory, the deflection state equations in the two deformation processes are obtained so that the stress–strain state can be determined, and the critical fault displacement for the transformation of pipe–soil coordinated and non-coordinated deformation is given, which can be used to evaluate the state of pipe–soil interaction. Although the stress state of the pipeline under soil backfilling can be obtained through the deflection curve equation of the pipeline, soil cannot protect the pipeline from failure under fault displacement due to its limitations. Therefore, a new backfill material with better bonding force with the pipeline, easy construction, and good protection for buried pipelines is urgently needed. Foam concrete (FC) is characterized by light-weight, controllable-strength, self-compacting, high-homogeneity and convenient-construction^23–25^, which provides a new choice for backfill engineering to control settlement or as an energy-absorbing material^26–28^.

With the in-depth study of the performance of FC, it has been widely used in backfilling engineering. Chen et al.^29^ examine the use of FC with cement partially replaced by fly ash for urban abandoned underground-space backfilling. Using FC as a backfill has negligible effects on surrounding structures, which proves that it is thus a promising material for underground-space backfilling. Wu et al.^30^ research the water stability property, dry shrinkage property, drying-wetting property, and freeze–thaw property of FC by mixing silt soil with foam concrete under conditions of different densities and silt soil content, the stability and durability of silt-based FC are considered outstanding and meet the needs of road engineering construction. Rezaei et al.^31^ investigate the strength, drying shrinkage, and thermal expansion coefficient of alkali-activated foams, and it can be used to backfill the narrow trench. Huang et al.^27^ propose using FC as subgrade filler, thereby controlling subgrade settlement in special soil areas.

Although FC is widely used in highway and municipal engineering, its application in the buried pipeline is still at an initial phase and the performance of FC in pipeline backfilling and pipeline protection has not been mastered yet. Therefore, the application of FC in the pipeline backfill project needs to be further studied. And in this paper, the pipe-FC interaction mechanism and the protective effect of FC on buried pipelines under active fault displacement were investigated. Firstly, the mechanism of the pipe-FC interaction is compared with that of the soil through theoretical analysis, and the stress states of the pipeline whose trench is backfilled with FC are determined, which theoretically proves the protective effect of FC. Then, the mechanical properties of FC were obtained through experiments. Finally, taking the cross-normal fault pipeline as an example, a FE model of the foam concrete-pipeline-soil interaction system is established by Abaqus to study the protective effect of FC on pipelines. The results show that the trench backfilled with FC can significantly reduce the pipeline strain under normal fault displacement, which provides a reference for the application of FC in trench backfilling and the protection of buried pipelines across faults.

## Pipe–soil interaction mechanism

**Figure 1:**
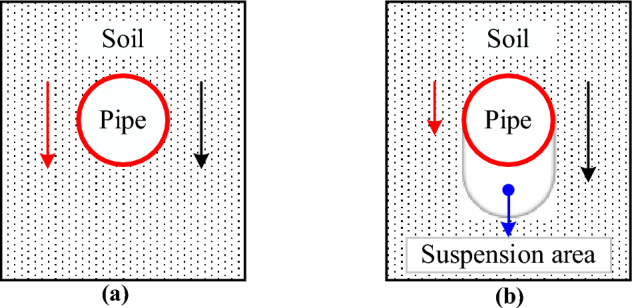
Schematic diagram of pipe–soil interaction. (**a**) Pipe-soil coordinated deformation, (**b**) Pipe-soil non-coordinated deformation.

Determining the mechanism of the pipe–soil interaction is the key to obtaining the stress state of the buried pipeline crossing the fault. The pipe–soil interaction changes gradually with the increase of the fault displacement (*δ*)^32–35^. Take the normal fault as an example, whose hanging wall moves downward relative to the foot wall. The pipeline sinks together with the soil under the soil pressure and the pipeline self-weight when *δ* is small, and the pipe–soil interaction shows coordinated deformation, as shown in Fig. 1a. Because of the greater deformation stiffness of the pipeline, its deformation will be smaller than that of the soil as the *δ* increases, and the soil counterforce below the pipeline will gradually decrease. When the soil counterforce reduces to 0, the pipeline will be out of contact with the soil^36–38^. With the *δ* further increase, there will be a suspended area below the pipeline. At this time, the pipe–soil interaction shows non-coordinated deformation, as shown in Fig. 1b.

The buried pipeline will be subjected to different loads under different pipe–soil deformations, including the self-weight of the pipeline and medium and the above soil pressure and the soil counterforce. The friction between pipeline and soil is ignored because of the protective layer wrapped around the pipeline and the slow rate of soil deformation during the fault movement. Assuming that the soil pressure is uniform, the load on the pipeline is:

1 $$q = q_{m} + q_{f} + q_{v}$$

2 $$q_{m} = \frac{\pi }{4}\rho_{m} g(D^{2} - d^{2} )$$

3 $$q_{f} = \frac{\pi }{4}\rho_{f} gd^{2}$$

4 $$q_{v} = \rho_{v} ghD$$

where *q*: Total load, N; *q*_*m*_: Pipeline self-weight, N; *q*_*f*_ : Medium self-weight, N; *q*_*v*_: Soil pressure above the pipeline, N; *ρ*_*m*_: Pipeline density, kg/m^3^; *ρ*_*f*_: Medium density, kg/m^3^; *ρ*_*v*_: Soil density, kg/m^3^;* h*: Burial depth of the pipeline, m; *D*: Pipeline outer diameter, m; *d*: Pipe inner diameter, m.

**Figure 2:**
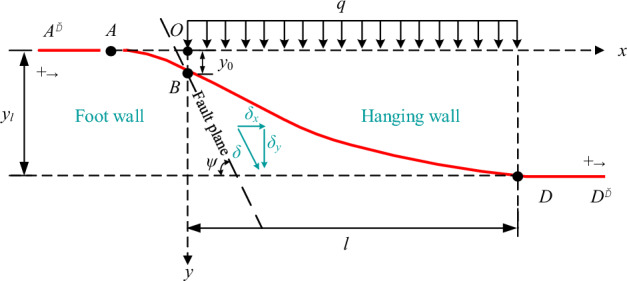
Mechanical model of pipeline under soil backfill.

The fault movement is regarded as a quasi-static process, so the loading of the pipeline is always in equilibrium. Based on the simplified loading, the large deformation area of the pipeline is taken as the research object, and the mechanical model (Fig. 2) of the pipeline is established whose trench is backfilled with soil under the normal fault displacement^13,14,39,40^. Take the intersection of the undeformed pipeline and the fault plane as the coordinate origin *O* to establish the coordinate system, as shown in Fig. 2. The pipeline section *x* = 0-*l* is regarded as a semi-infinite continuous elastic foundation beam to analyze the stress state of the pipeline under the pipe–soil coordinated and non-coordinated deformation.

### Pipe–soil coordinated deformation

The principle of elastic foundation beam theory^41,42^ can be utilized to comprehend the stress distribution of pipelines near fault zones and the bearing capacity of soil on the pipelines. When the interaction between the pipeline and soil exhibits coordinated deformation, based on the elastic foundation beam theory, the pipeline and the surrounding soil are considered as an integrated structure, with shear deformation neglected. The governing differential equation for this section can be expressed as follows:

5 $$EI\frac{{d^{{4}} y}}{{dx^{{4}} }} + ky = q$$

where *E*: Pipeline elastic modulus, MPa; *I*: Pipeline section moment of inertia, m^4^; *x*: Distance to *O*, m; *y*: Pipeline deflection, m; *k*: Elastic foundation coefficient; *q*: Loads on the pipeline.

Equation (5) is a fourth-order linear non-homogeneous differential equation with constant coefficients, and its solution is composed of the general solution of the homogeneous equation and four special solutions. The corresponding homogeneous equation of Eq. (5) is:

6 $$EI\frac{{d^{{4}} y}}{{dx^{{4}} }} + ky = 0$$

Let $\alpha = \sqrt[4]{\frac{k}{4EI}}$, and the solution of Eq. (6) is:

7 $$y = A_{1} ch\alpha x\cos \alpha x + A_{2} ch\alpha x\sin \alpha x + A_{3} sh\alpha x\cos \alpha x + A_{4} sh\alpha x\sin \alpha x$$

It is considered that the pipeline load in section *x* > 0 is distributed uniformly, and the integral boundary is (0, *x*), then the four special solutions of the Eq. (5) can be written as:

8 $$\left\{ {\begin{array}{*{20}l} {\Delta y = \frac{q}{k}(1 - \varphi_{1} )} \hfill \\ {\Delta \theta = \frac{2\alpha }{k}(\varphi_{2} - \varphi_{3} )} \hfill \\ {\Delta M = - \frac{q}{{2\alpha^{2} }}(\varphi_{4} )} \hfill \\ {\Delta Q = - \frac{q}{2\alpha }(\varphi_{2} + \varphi_{3} )} \hfill \\ \end{array} } \right.$$

Note: *φ*_1_ = ch*αx*cos*αx*, *φ*_2_ = ch*αx*sin*αx*, *φ*_3_ = sh*αx*cos*αx*, *φ*_4_ = sh*αx*sin*αx.*

Therefore, when the pipeline and soil experience coordinated deformation, the deflection curve equation of the pipeline can be expressed as Eq. (9), which can be used to determine the stress state of the pipeline.

9 $$y_{1} = A_{1} \varphi_{1} + A_{2} \varphi_{2} + A_{3} \varphi_{3} + A_{4} \varphi_{4} + \Delta y + \Delta \theta + \Delta M + \Delta Q$$

### Pipe–soil non-coordinated deformation

When the coordinated deformation between pipeline and soil reaches the critical state, the soil counterforce reduces to 0, *ky* = 0, and the pipe–soil interaction enters the non-coordinated deformation. The governing differential equation of the pipeline section *x* = 0-*l* can be expressed as:

10 $$EI\frac{{d^{{4}} y}}{{dx^{{4}} }} = q$$

Then the deflection curve equation of the pipeline can be written as:

11 $$y_{2} = B_{1} x^{4} + B_{2} x^{3} + B_{3} x^{2} + B_{4} x^{1} + B_{5}$$

With the increase of *δ*, the soil counterforce in *x* < 0 gradually increases, while that of in* x* > 0 gradually decreases, which can be inferred that the pipeline at *x* = 0 is the first position to detach from the soil. When *δ* reaches *δ*^crit^, that is, the critical displacement of the pipe–soil transformation from coordinated to non-coordinated deformation, the pipeline at *x* = 0 reaches the limit of coordinated deformation. To solve the Eq. (11), the boundary condition at *x* = *l* is taken as:

12 $$\left\{ {\begin{array}{*{20}c} {y_{l} = - \delta_{y} } \\ {\theta_{l} = 0} \\ {M_{l} = 0} \\ {Q_{l} = 0} \\ \end{array} } \right.$$

By combining Eqs. (10) to (12), the deflection curve of the *x* = 0-*l* can be solved as Eq. (13), which can be used to solve the stress state of the pipeline.

13 $$y_{2} = \beta x^{4} - 4\beta lx^{3} + 6\beta l^{2} x^{2} - 4\beta l^{3} x - \delta + \beta l^{4}$$

Note: $$\beta = \frac{q}{24EI}$$.

### Transformation critical fault displacement ***δ***^crit^

The stress state of the pipeline under the pipe–soil coordinated deformation is different from that under the pipe–soil non-coordinated deformation. The contact state of the pipeline and soil can be determined by the *δ*^cirt^, and then the stress state of the pipeline can be obtained according to its corresponding deflection curve equation.

According to Eq. (13), the stress state at *x* = 0 can be obtained as:

14 $$\left\{ {\begin{array}{*{20}c} {y_{0} = - \delta_{y} + \beta l^{4} } \\ {\theta_{0} = - 4\beta l^{3} } \\ {M_{0} = 12\beta l^{2} } \\ {Q_{0} = 24\beta l^{2} } \\ \end{array} } \right.$$

When *δ* = *δ*^crit^, the pipeline deformation at *x* = 0 meets both the Eqs. (6) and (10) at the same time, so the Eq. (14) can be used as the boundary condition of the Eq. (7). The equation of the pipeline deflection curve at the critical state of the pipe–soil coordinated deformation can be written as:

15 $$y_{1} = y_{0} \varphi_{1} + \theta_{0} \varphi_{2} + M_{0} \varphi_{3} + Q_{0} \varphi_{4} + \Delta y + \Delta \theta + \Delta M + \Delta Q$$

Then, the *δ*^cirt^ is solved as Eq. (16) based on *y*_1_|_*x*=*1*_ = *y*_2_|_*x*=*1*_.

16 $$\delta^{{{\text{crit}}}} = \frac{{\beta l^{4} \varphi_{1} |_{x = l} + \theta_{0} \varphi_{2} |_{x = l} + M_{0} \varphi_{3} |_{x = l} + Q_{0} \varphi_{4} |_{x = l} + \Delta y|_{x = l} + \Delta \theta |_{x = l} + \Delta M|_{x = l} + \Delta Q|_{x = l} }}{{(\varphi_{1} |_{x = l} - 1)\sin \psi }}$$

When the pipe–soil interaction enters the non-coordinated deformation, the self-weight of the pipeline and medium and the above soil pressure is borne by the pipeline alone. Loads on the pipeline increase significantly, and the pipeline will be more likely to be damaged. Therefore, the pipe–soil interaction should be avoided to enter the non-coordinated deformation.

However, it is easy for the pipeline and soil to experience the non-coordinated deformation under the *δ* because of the soil looseness. Therefore, it is necessary to avoid the non-coordinated deformation by choosing the material with a better bond force. Foam concrete (FC) can be used as backfill material for the pipe trench due to its good wrapping, low self-weight, easy construction, and maintenance.

## Pipe-FC interaction mechanism

FC is a lightweight material composed of cement paste and a certain proportion of tiny stable bubbles, resulting in the characteristics of lightweight, controllable strength, high-flowability, high-homogeneity, convenient-construction, and negligible influence on the environment. To determine the pipe-FC interaction mechanism, the mechanical properties and the constitutive of FC are studied through the experiment.

### Mechanical properties and constitutive model of FC

A uniaxial compression test of FC was conducted to study the mechanical properties of FC. Meanwhile, a comparative analysis was carried out with the experiments of ref.^43^ to ensure the reliability of the experimental results in this paper.

The strength of FC is mainly provided by cement. Based on the Technical Specification for Foamed Mixture Lightweight Soil Filling Engineering CJJ/T177-2012(Chinese Standard)^44^, ordinary Portland cement with a strength grade of 42.5 was selected in this experiment. The foam used in this experiment was prepared with industrial hydrogen peroxide^43,45^.

**Figure 3:**
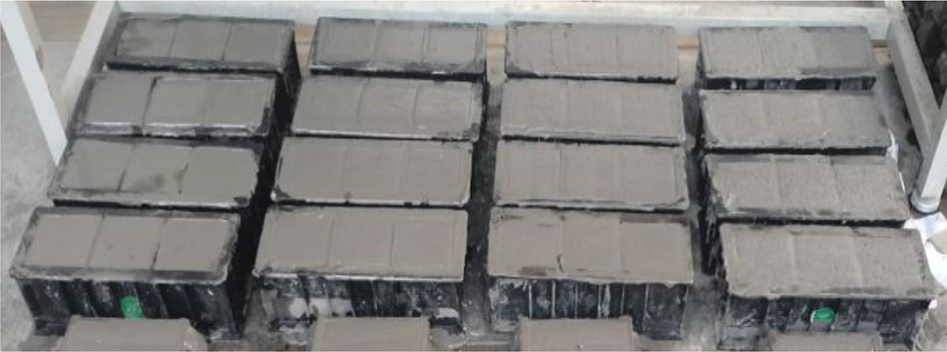
The mold of foam concrete.

**Figure 4:**
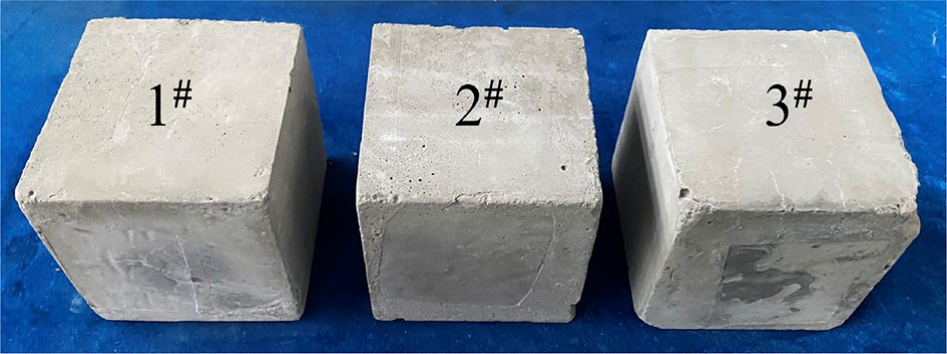
Foam concrete specimens.

During the preparation of FC specimens, the water-cement ratio was guaranteed to be 0.5^41,46^. After mixing the cement with the foam, the forming uniform paste was immediately poured into the 100 mm × 100 mm × 100 mm triple test mold (Fig. 3). After storing in the natural environment for three days, took out all specimens and put them into a standard curing box with a temperature of 20℃ and relative humidity of over 90% for curing 28 days. The FC specimens were prepared with three densities of *ρ* = 400 kg/m^3^, *ρ* = 600 kg/m^3^, and *ρ* = 800 kg/m^3^ (Fig. 4).

**Figure 5:**
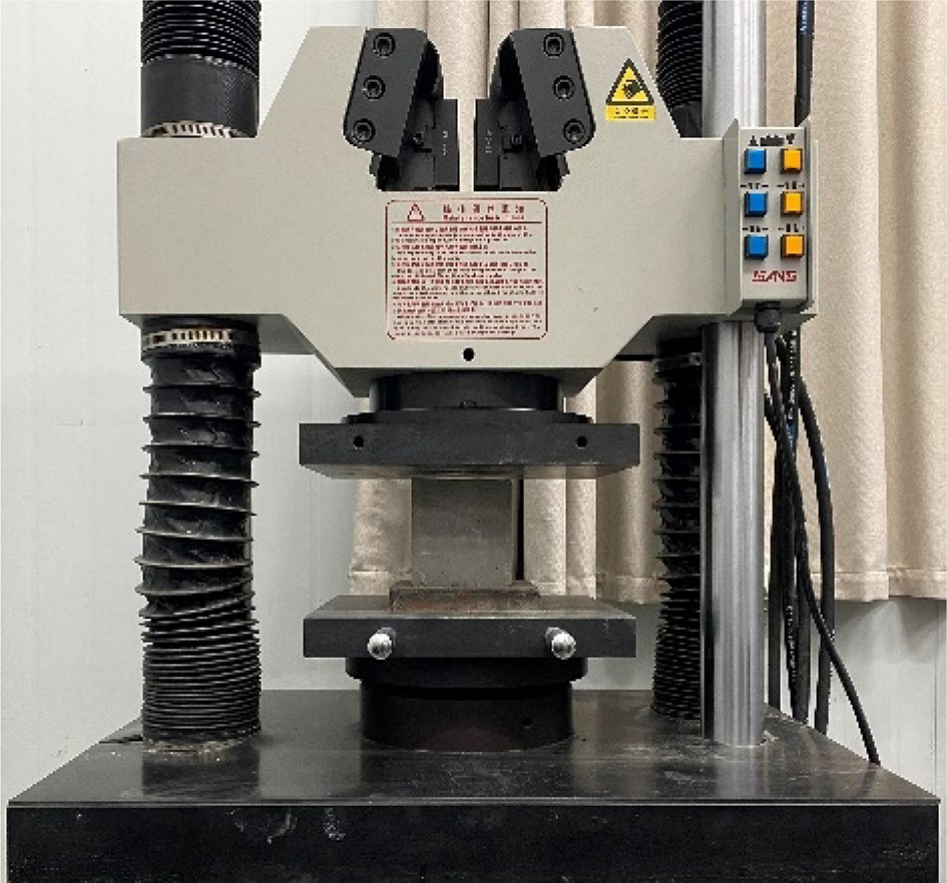
Experimental test equipment.

**Figure 6:**
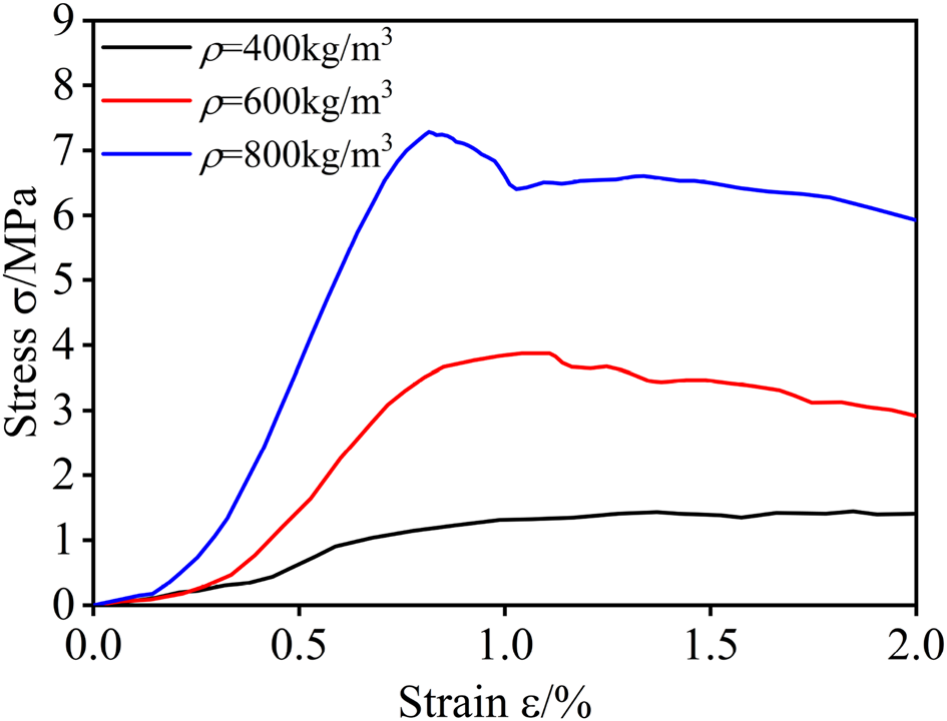
The uniaxial compression stress–strain curves of FC with various densities.

It can be known that FC has low tensile strength, showing obvious brittleness and the plastic damage can be neglected, referring to the experimental results of ref.^43^. The ultimate tensile strength of FC is about 10% of its ultimate compressive strength. Therefore, this paper does not pay attention to the tensile strength and damage of FC. Figure 5 shows that the uniaxial compression test was carried out on the universal testing machine. Each density of FC was selected in three groups containing three specimens, and the average compressive stress–strain curves of the above three densities were obtained, as shown in Fig. 6.

From Fig. 6, we can see that at the initial loading stage, the stress of FC increases linearly with the increase of strain. The load is mainly borne by the elastic deformation of FC. With loading, the cell wall collapses and cracks appear inside the specimen, and the stress increases nonlinearly with the increase of strain until it reaches the peak. With the continuous loading, the micro-cracks in the specimen develop continuously and eventually develop into through cracks, leading to the destruction of the specimen and the reduction of the bearing capacity.

The uniaxial compressive stress–strain curves of FC and ordinary concrete are similar. However, the FC specimen still has a certain bearing capacity after reaching the ultimate compressive strength compared with ordinary concrete, which shows the apparent ductility under compressive load. The ductility of FC also provides the feasibility for its application as backfill material for buried pipeline trench. The compressive stress–strain curves of FC obtained by experiments are similar to ref.^43^.

**Figure 7:**
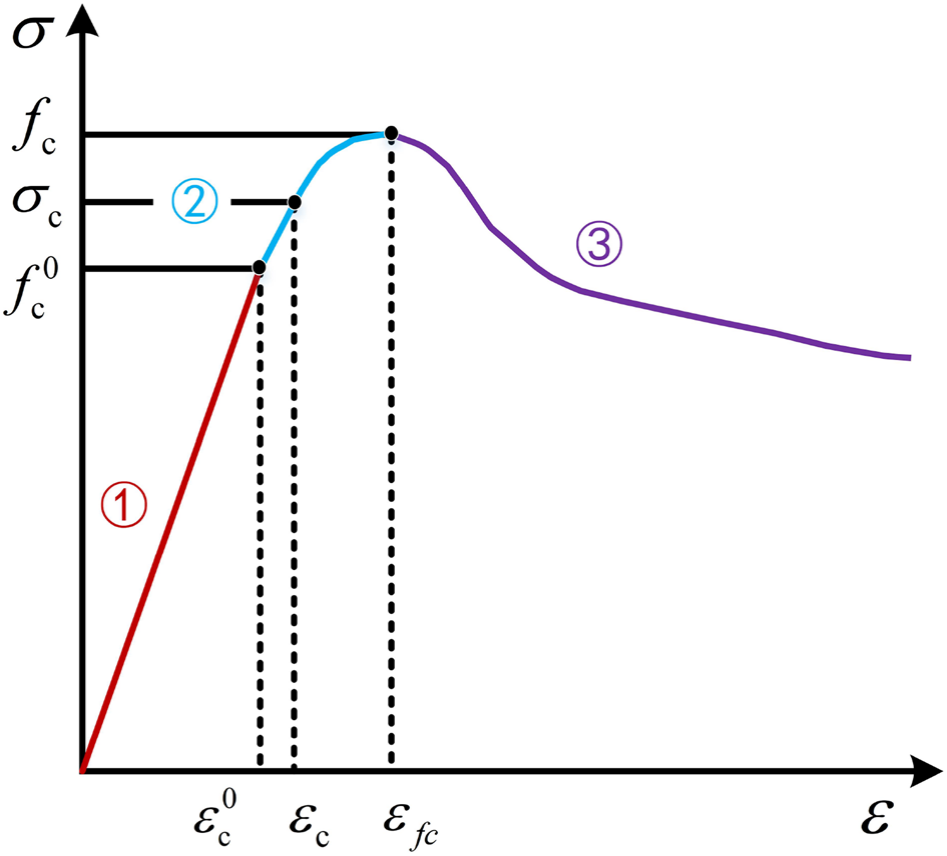
Compression constitutive model of FC.

Based on the experimental data obtained from uniaxial compression tests of FC specimens with different densities, the compressive constitutive model of FC is presented in Fig. 7. Under uniaxial compression, the yield strength of FC has not been clearly defined. Therefore, the strain offset method is used to define the compressive yield strength of FC in this study. Some studies have selected different strain offset values for different porous materials^47,48^. The strain offset value of 0.02% was chosen in this paper to determine the yield strength of FC^43^.

According to the elastic stress *f*_c_^0^ and strain *ε*_c_^0^, yield stress *σ*_c_ and strain *ε*_c_, ultimate stress *f*_c_ and strain *ε*_*f*c_, the uniaxial compression curve of FC can be divided into ①elastic stage (*ε* < *ε*_c_^0^), ②plastic stage (*ε*_c_^0^ < *ε* < *ε*_*f*c_) and ③damage stage (*ε* > *ε*_*f*c_). Therefore, the mechanical properties parameters of FC were obtained.

### Pipe-FC coordinated deformation

FC becomes a whole with the pipeline after the trench is backfilled with FC, and the pipeline load state changes relative to that of backfilled with soil. When the hanging wall moves downward relative to the foot wall, the FC trench on the side of the hanging wall will be separated from the soil. The FC trench and the soil show non-coordinated deformation, but the FC and the pipeline show coordinated deformation. The pipeline and FC can bear the load (*Q*) together, which includes the self-weight of the pipeline, medium, and FC.

**Figure 8:**
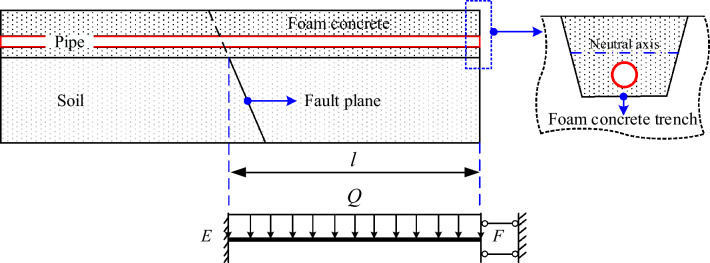
Mechanical model of pipeline under FC backfill.

The mechanical model of the pipeline under FC backfill is established by simplifying the load to obtain the pipe-FC interaction. The FC and the pipeline on the hanging wall side are regarded as a beam subjected to uniform load *Q* (shown in Fig. 8). The boundary conditions of *E* and *F* are a fixed end and vertical sliding support, respectively.

Based on the mechanical model, the bending moment at the end of *E* and *F* are calculated as *M*_E_ = *Ql*^2^/3 and* M*_F_ = *Ql*^2^/6, respectively. Therefore, the bending moment of the FC trench at *E* is the largest, and its stress can be expressed as *σ* = *M*_E_*y*/*I*.

Due to the low tensile strength of FC, cracks will inevitably appear in the tensile zone of the FC trench during the fault movement, but the cracks cannot be penetrated, the pipeline and the FC trench compression zone can still bear the load together, and the appearance of cracks does not affect the coordinated deformation between the pipeline and FC. Due to the characteristics of FC, it can be used as a backfill material for the pipeline trench to reduce pipeline stress and strain. However, the protective effect of FC compared with soil cannot be expressed quantitatively by theoretical analysis. Although the experiment results are convincing, some limitations are not representative and universal. Additionally, it is time-consuming and laborious to conduct full-scale experiments to study the effect of FC on buried pipelines crossing normal fault. So it is necessary to quantitatively analyze the pipeline stress state under the soil and the FC backfilling through numerical simulation (by using Abaqus) to determine the protective effect of FC.

### Numerical simulation of FC

To guarantee the accuracy of the numerical simulation, the correct choice of FC constitutive model in Abaqus is essential.

There are many concrete plastic models in Abaqus, among which CDP (Concrete Damaged Plastic Model) can be used in unidirectional loading, cyclic loading, and other occasions with good convergence. Thus, the CDP model is adopted as the plastic model of FC in this paper. The tensile plastic damage of FC is not considered because of its tensile brittleness. Meanwhile, on the basis of the experimental and the reference experimental results, the stress–strain curves of FC are similar to those of ordinary concrete. Therefore, in the CDP model, the parameters of Plasticity refer to ordinary concrete settings: expansion angle *Ψ* = 30°, flow potential offset value *m* = 0.1, ratio of biaxial ultimate compressive strength to uniaxial ultimate compressive strength *α*_*f*_ = 1.16, ratio of second stress invariants on the tensile meridian plane and compression meridian plane *γ* = 0.6667, and viscosity coefficient *μ* = 0.

**Figure 9:**
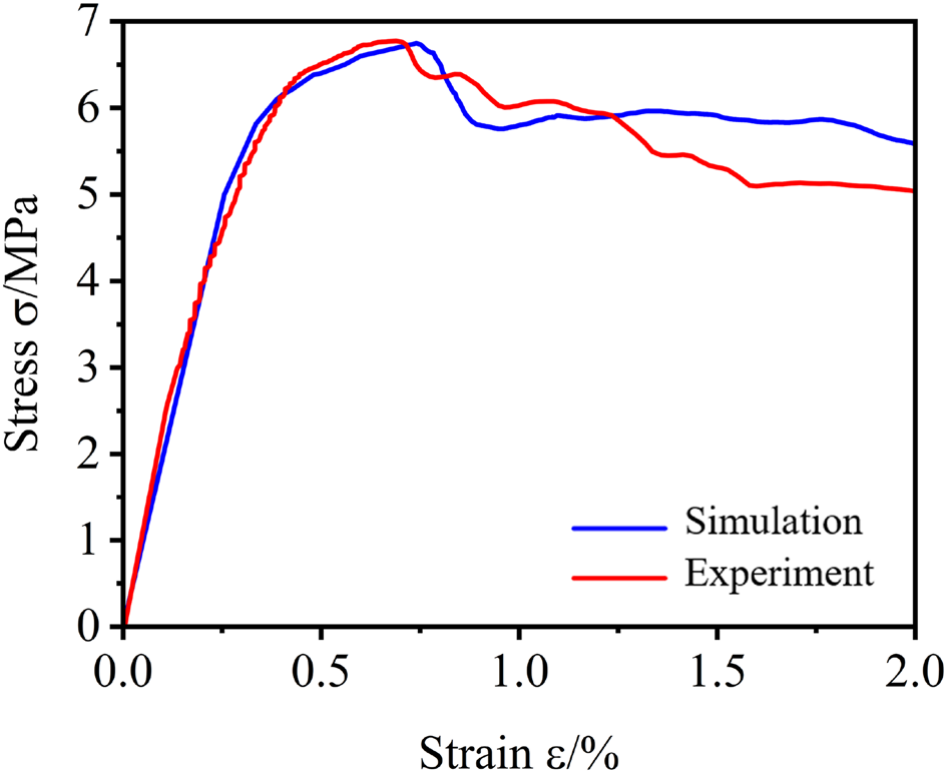
Constitutive model relationship between simulation and experiment.

As shown in Fig. 9, the simulated data of FC are in good agreement with those of experimental. The CDP model of the software accords with the stress–strain law of the FC, which can guarantee the accuracy of the simulation result of FC. Therefore, the full-scale experiment can be replaced by numerical simulation.

## FE model of pipeline crossing normal fault

**Figure 10:**
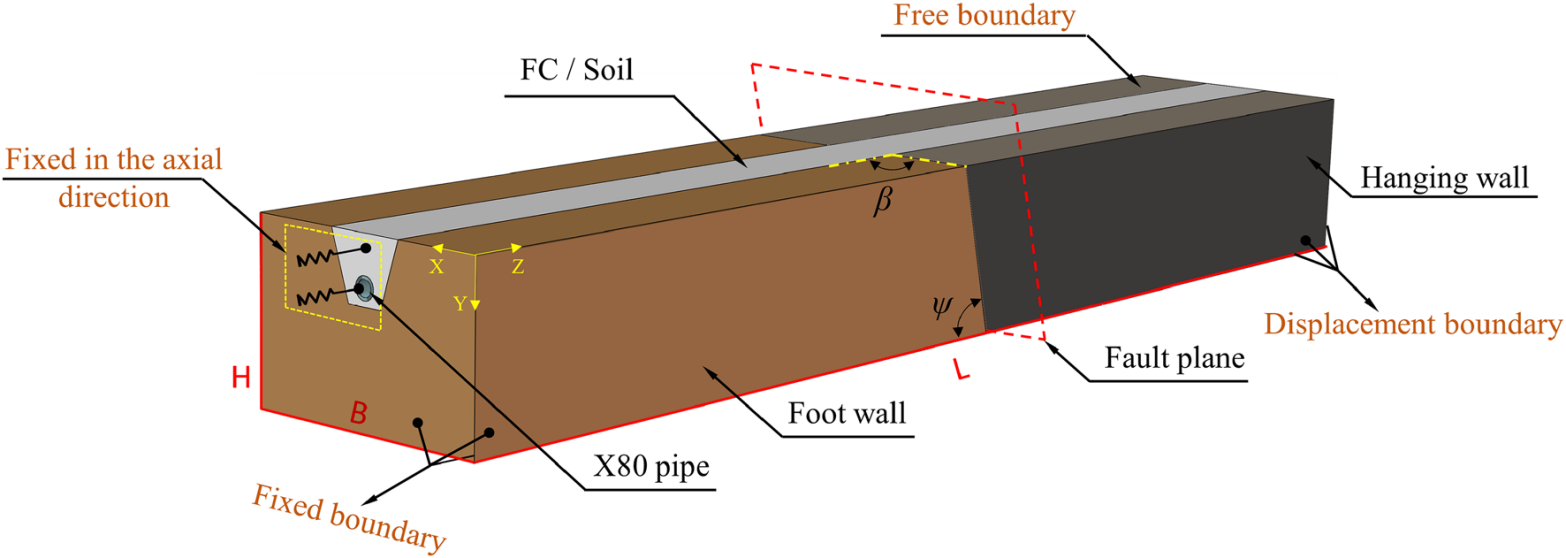
Finite element model.

Aiming at the semi-infinite domain problem of long-distance oil and gas pipelines across faults in numerical simulation, this paper focused on analyzing the pipeline-FC / soil interaction module with large deformation and torsional response near the fault. Based on the research of the American Society of Mechanical Engineers (ASME) and Vazoursa et.al.^11^, when the dimensions of the model in X, Y, Z directions are 11*D*, 8*D*, and 65*D* respectively (*D* is the pipe diameter), that is, *H* = 10 m, *B* = 15 m, and *L* = 80 m, which can meet the accuracy requirements of the pipeline strain. Meanwhile, S4R elements were used to simulate the pipeline, while C3D8R elements simulated the FC, soil, and rock in Abaqus. It should be note that, in order to effectively investigate the influence of fault displacements on the interaction between pipelines and foam concrete, while enhancing computational efficiency, this study adopts a model referenced in Refs.^14,39,40,49,50^ to develop a simplified approach disregarding seismic fault zone thickness, aiming to simulate fault displacements under seismic conditions. In addition, to simplify the numerical simulation process, the end of the pipeline and FC on the foot wall was constrained in the axial direction, while the other end of the pipeline and FC were unconstrained and could move with the hanging wall^13^. The established numerical model is shown in Fig. 10.

**Figure 11:**
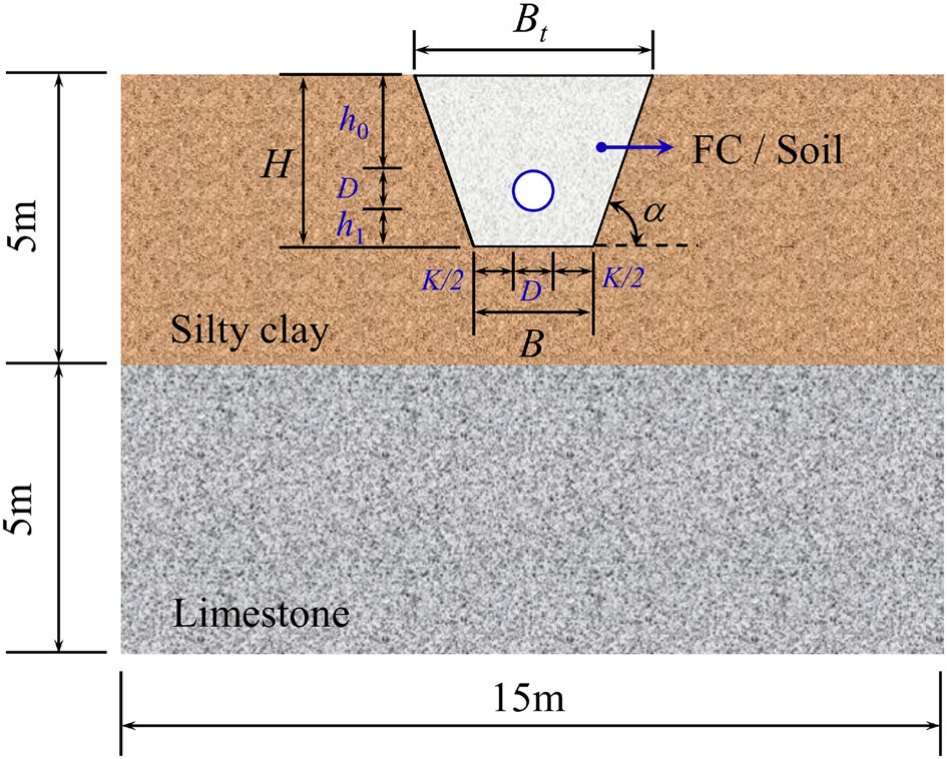
Size of FE model cross-section and pipe trench.

Based on the Design of Gas Transmission Pipeline Engineering GB50251-2015 (Chinese Standard)^21^, when the native soil is silty clay, and there is no load on the slope top, in order to prevent slope from collapsing, the maximum trench slope ratio is tan*α* = 3.03 and the bottom cushion thickness *h*_1_ = 0.3 m, the burial depth of pipeline *h*_0_ = 1.2 m. Meanwhile, the bottom of the trench needs to be widened and excavated to ensure the welding and laying working face, whose size can be calculated by Eq. (17). The cross-sectional size of the FE model and the trench is shown in Fig. 11.

17 $$B = D + K$$

where *B* is the bottom width of the trench, m; *B*_t_ is the top width of the trench; *K* is the allowance for trench bottom widening (convenient for construction), m; When the trench depth *H* < 3 m, *K* = 0.8 m; When the trench depth 3 < *H* < 5 m,* K* = 1.0 m.

### Material model

The X80 steel pipeline with diameter *D* = 1219 mm and wall thickness *t* = 22 mm is selected as the research object. The Ramberg–Osgood model, whose stress–strain relationship can better meet actual pipe material, is adopted for the pipe constitutive, and the constitutive expression is shown in Eq. (18). The X80 pipeline has high toughness, which can bear large tensile stress and strain. Therefore, the compression failure of the pipeline generally precedes the tensile failure of the pipeline under the action of fault displacement^14^, and its critical compressive strain can be calculated as *ε*_c_^crit^ = 1% according to the code CSA-Z662:19^51^.

18 $$\varepsilon = \frac{\sigma }{E}\left[ {1 + \frac{\alpha }{1 + r}\left( {\frac{\sigma }{{\sigma_{s} }}} \right)^{n} } \right]$$

where *ε* is the strain of the pipeline, MPa;* σ* is the stress of pipeline MPa; *σ*_*s*_ is the yield stress of the pipeline, MPa; *E* is the elastic modulus, MPa; *n* is the hardening coefficient; *α* and *r* are parameters of Ramberg–Osgood model.

Considering the influence of the underlying rock stratum on the pipeline strain in the engineering, the stratum within 5m above the ground is silty clay (SC), while the stratum 5m below is limestone, illustrated in Fig. 11. The Mohr–Coulomb model is used as the constitutive model of strata^2,52–54^, and the strata parameters are shown in Table 1.

**Table 1:** Physical parameters of native and backfill materials.

Material	*ρ* (kg/m^3^)	*E* (MPa)	*μ*	*c* (kPa)	*φ* (°)	*f*_c_ (MPa)
Silty clay (SC)	1900	33	0.27	35	22	None
Limestone	2090	28,500	0.29	6720	42	None
Soil	2000	30	0.3	10	35	None
Foam concrete (FC)	800	820	None	None	None	7.5

Because of the low density of FC, the buoyancy of groundwater should be taken into account when backfilling the buried pipeline trench with FC. As a result, the density of FC should be greater than that of water. However, the working conditions considered in this paper have no groundwater. Moreover, the pipeline density is much higher than that of water. Therefore, the FC with *ρ* = 800 kg/m^3^ was selected to backfill the trench in consideration of the cost of FC.

### Interaction model

**Figure 12:**
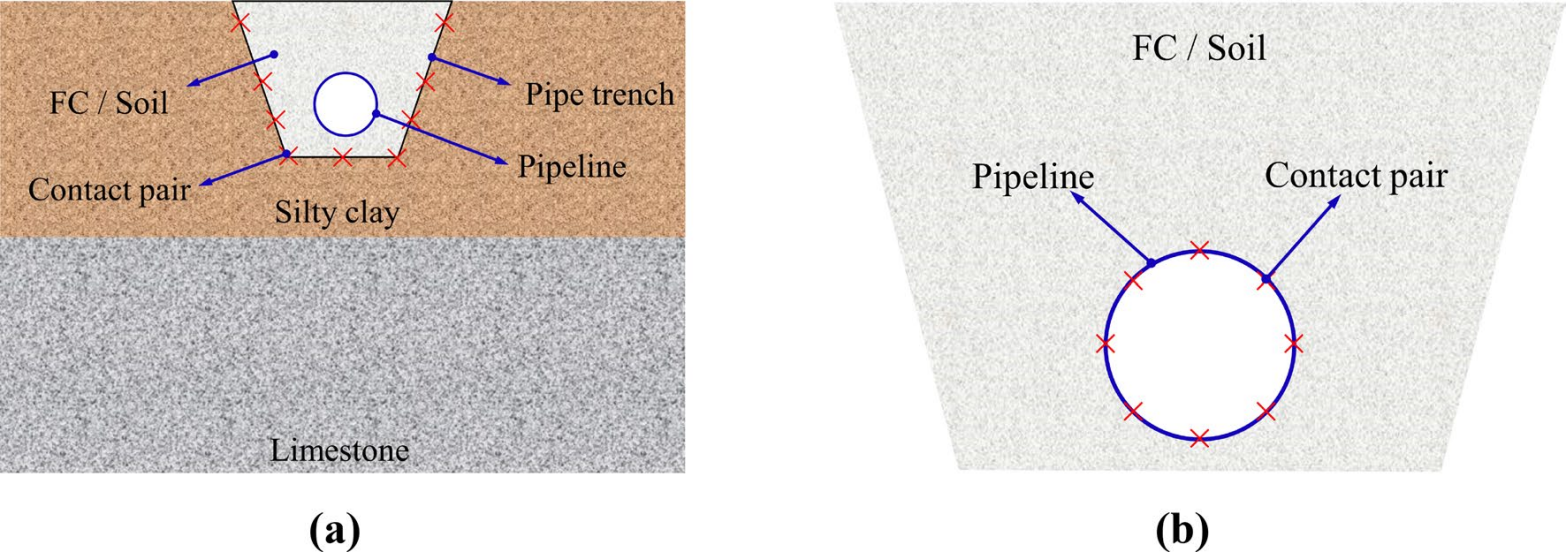
Schematic diagram of interaction contact model.

Considering the material nonlinearity of the pipeline, FC, SC, and soil, and the state nonlinearity caused by large geometric deformation, the FC/soil-SC (Fig. 12a) and pipeline-FC/soil (Fig. 12b) interaction were realized by the master–slave contact algorithm in Abaqus. The outer surface of the material with high rigidity is taken as the target surface (rigid-body), and the flexible material surface with low rigidity is taken as the contact surface (deformable-body). The contact pair is constructed by establishing the target and contact element, and then the force between the contact surfaces is obtained by a nonlinear contact algorithm. The Mohr–Coulomb friction model transfers the shear stress to the contact surface, and the hard contact is used for normal action. The different coefficients of friction (*μ*) for different interacting surfaces, including pipeline-soil, pipeline-FC, and FC/soil-SC were 0.4, 0.6, 0.6 respectively^13,14^.

## Strain state of the pipeline under normal fault

The active fault displacements are generally caused by the earthquake. Therefore, the single and composed fault displacements *δ* can be determined by referring to the empirical statistical Eqs. (19) and (20) of engineering design in China and the relationship between earthquake intensity and magnitude^55^.

19 $$\lg \delta = - 3.019 + 0.4646M$$

20 $$I = 0.24 + 1.29M$$

where *δ* is the fault displacement, m; *M* is the earthquake magnitude;* I* is the earthquake intensity; Most pipelines in the seismic intensity area of VIII-X are easy to fail^5,6^, so the range of *δ* can be determined as 0.59 m-3.13 m.

Pipe-fault crossing geometry, crossing angle *β* and fault dip angle *ψ*, is the primary parameter affecting the pipeline mechanical behavior. According to refs.^56–58^, When *β* = 90°, the axial strain of the pipeline under the reverse fault component is the largest, and the pipeline is most prone to failure, thus, *β* = 90° was taken. The parameter *ψ* cannot be controlled, so *ψ* was taken as a variable, and *ψ* = 30°, 60°, 90°are selected to evaluate the protective effect of FC for the pipeline. High-grade, large-diameter pipelines can withstand larger stress and strain and can operate in the plastic zone. Therefore, this study use the strain evaluation criteria to assess the condition of pipe wall^21,59^.

### Trench backfilled with soil

**Figure 13:**
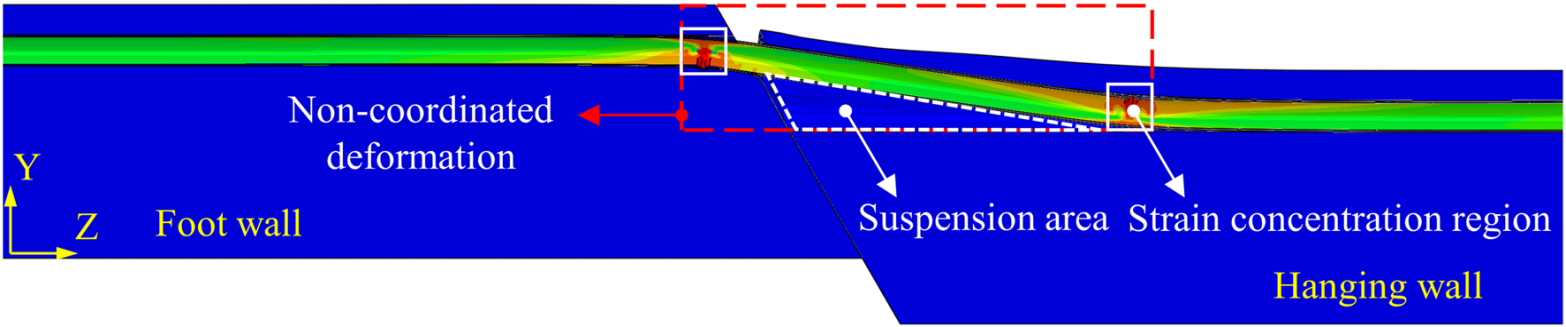
Deformation diagram of the pipeline and soil under normal fault *δ* = 3 m.

Under the normal fault displacement, the hanging wall moves downward relative to the foot wall, and the soil trench induces compression on the pipeline, forcing the pipeline to bend, thus causing the pipeline to be subjected to tensile and compressive stress. Taking the normal fault with *ψ* = 60° as an example, the deformation of the pipeline and soil is illustrated in Fig. 13. Due to the difference in deformation between the pipeline and soil, there are coordinated and non-coordinated deformation areas in pipe–soil interaction. Moreover, the strain concentration occurs in the pipeline at the junction of the two areas, which may result in pipeline failure.

**Figure 14:**
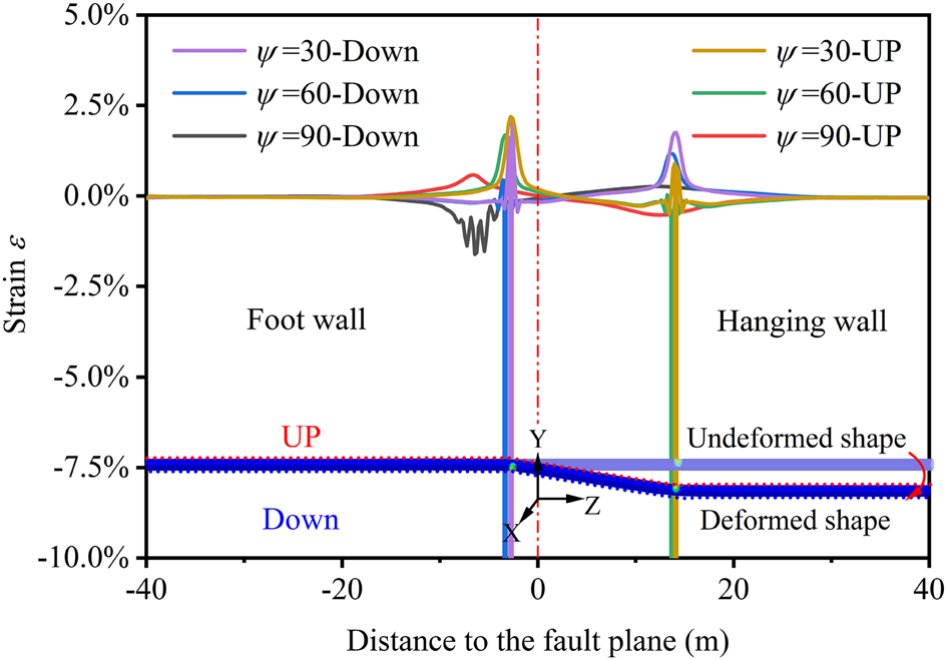
Strains at different positions of pipelines under normal fault *δ* = 3 m with *ψ* = 30°, 60°, 90°

Figure 14 depicts the strain at different pipeline positions, concluding that severe strain concentration occurs in the pipeline on the hanging and foot wall sides. Meanwhile, the peak tensile and compressive strains of the pipeline on the foot wall are larger. With the increase of *ψ*, the vertical displacement of the normal fault increases, and the peak tensile and compressive strain of the pipeline increases gradually. The pipeline compressive strain reaches more than10%, far exceeding the critical compressive strain and threatens pipeline safety. When *ψ* = 90°, the failure to the pipeline is the greatest, and the maximum strain position of the pipeline has not changed obviously.

### Trench backfilled with FC

**Figure 15:**
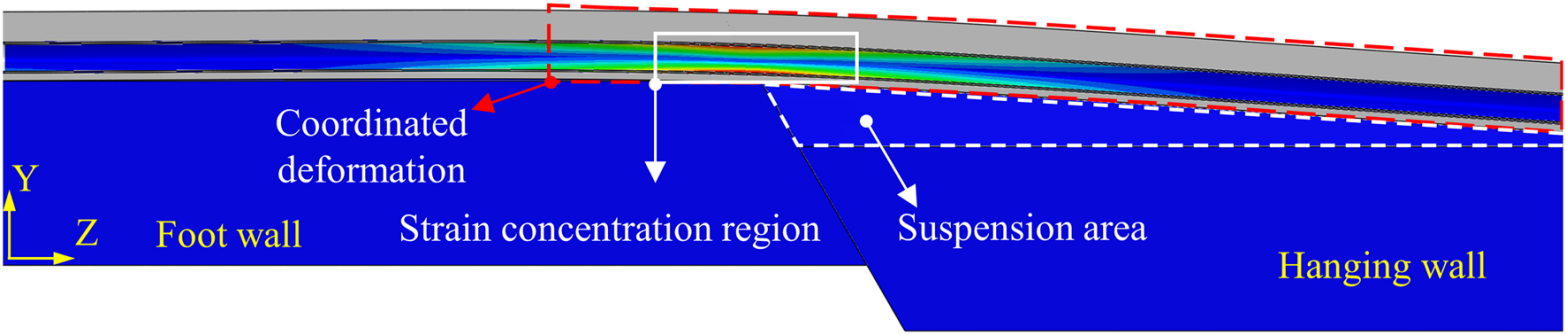
Deformation diagram of the pipeline and FC under normal fault *δ* = 3 m.

Taking the normal fault with *ψ* = 60° as an example, the deformation diagram of the pipeline and FC is shown in Fig. 15. Although the FC trench is separated from the soil under *δ*, FC always keeps the coordinated deformation with the pipeline. With the increase of the *δ*, the stress concentration appears near the fault plane. The result shows agreeably to theoretical analysis of section “Pipe-FC coordinated deformation”.

**Figure 16:**
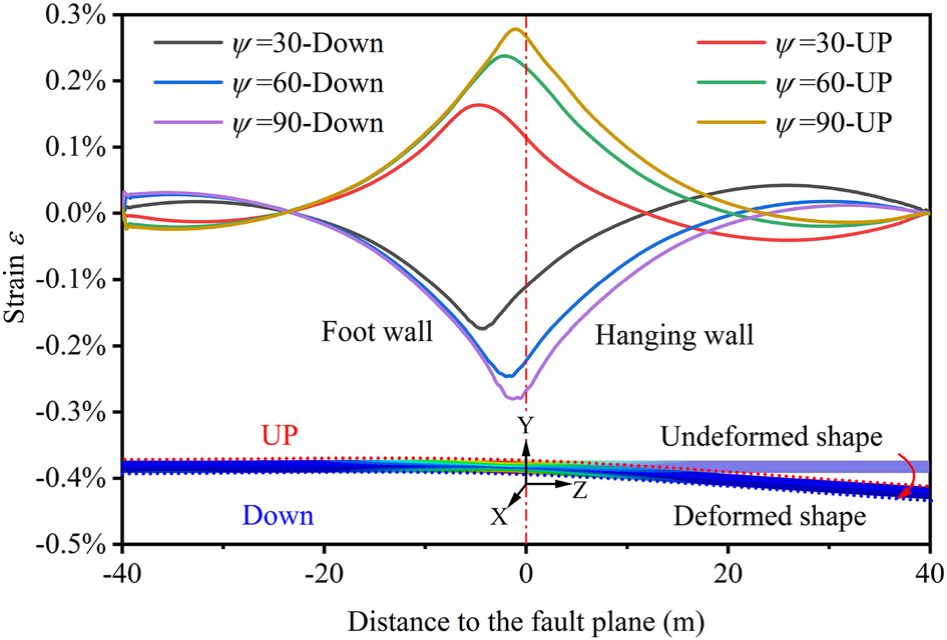
Strains at different positions of pipelines under normal fault *δ* = 3 m with *ψ* = 30°, 60°, 90°

The evolution of strains at different positions of pipelines (trenches backfilled with FC) under normal fault displacement with different *ψ* is illustrated in Fig. 16. We can see that peak strain positions of pipelines are near the fault plane, and with the increase of *ψ*, the pipeline peak strain increases. However, the peak tensile and compressive strains of the pipeline are all less than 0.3%, which can guarantee pipeline safety.

### Protective effect of the FC

The numerical simulation results in sections “Trench backfilled with soil” and “Trench backfilled with FC” prove that FC can decrease the pipeline strain when the pipeline is subjected to the normal fault displacement. Furthermore, the protective effect of the FC is analyzed quantitatively below by obtaining the strain evolution in the position of the strain concentration.

**Figure 17:**
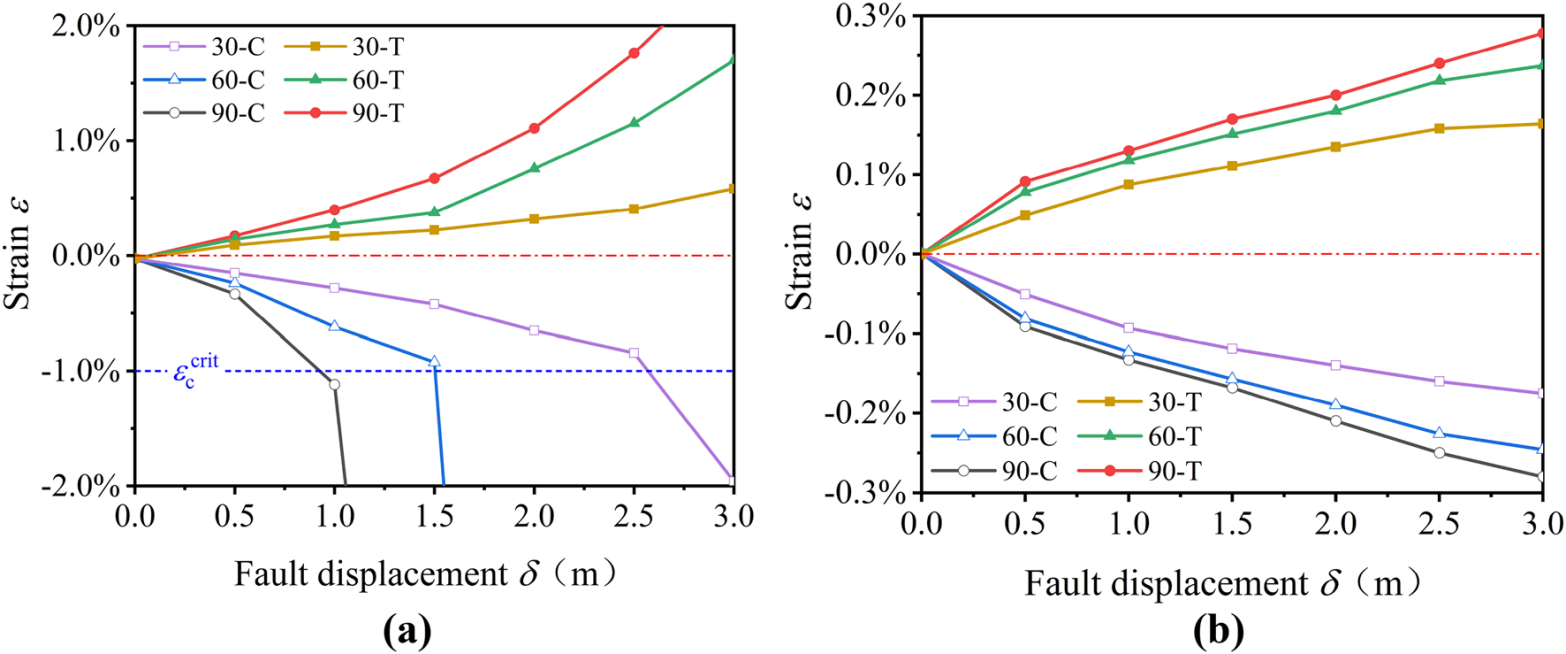
Evolution of tensile and compressive strains of pipelines; (**a**) trench backfilled with soil and (**b**) trench backfilled with FC under normal fault displacement with *ψ* = 30°, 60°, 90°.

Figure 17 shows the evolution of tensile and compressive strains of pipelines backfilled trenches with FC and soil subjected to the normal fault *δ* = 3 m with *ψ* = 30°, 60°, 90°, which can be concluded that the tensile and compressive strains increase with the increase of *ψ*. Figure 17a shows that the compressive strains of pipelines, where the trench are backfilled with soil, are reached *ε*_c_^crit^ easily under *δ* = 3 m. The pipelines have suffered a severe compressive failure. Meanwhile, with the increase of *ψ*, the tensile and compressive strains increase rapidly to 2.6% and 16%, respectively. In contrast, when the trenches are backfilled with FC, the pipeline tensile and compressive strains are less than 0.3% even under *δ* = 3 m (Fig. 17b).

The FC has a great protective effect on pipelines subjected to the normal fault displacement, and the protective effect of FC can be evaluated by the average strain reduction rate (SRR), as shown in Table 2. We can see that the average SSR obtained from compressive strains is 95.7%, while that obtained from tensile strains is 82.6%, so FC has a better protective effect on the pipeline subjected to compressive under the normal fault displacement.

**Table 2:** Strains of pipelines and the average SRR under normal fault *δ* = 3 m with different *ψ.*

Strain	Backfilled material	*ψ* = 30° (%)	*ψ* = 60° (%)	*ψ* = 90° (%)	Average SRR (%)
Tensile	Soil	0.58	1.70	2.60	82.6
	FC	0.16	0.24	0.27	
Compressive	Soil	2.00	12.00	16.00	95.7
	FC	0.18	0.25	0.28	

## Conclusions

In this paper, the pipe-FC interaction mechanism is studied and compared with the pipe–soil interaction mechanism under the normal fault displacement caused by the earthquake. Then, the FC-pipeline-soil interaction system is established by using Abaqus, and the numerical simulation results show that FC has a good protective effect on the pipeline under normal fault displacement, and the following conclusions are drawn:

1. The mechanism of the pipe–soil interaction is divided into coordinated and non-coordinated deformation, and their theoretical solutions are obtained. Meanwhile, the transformation critical displacement *δ*^crit^ between the coordinated and non-coordinated deformation is deduced, which can be used to analyze the state of the pipe–soil interaction
2. In terms of the deformation of FC and the pipeline, it is proved that FC can deform coordinated with the pipeline through theoretical analysis and numerical simulation, decreasing the strain concentration effectively. Therefore, FC can be used for buried pipeline trench backfilling to protect the pipeline under active fault displacement.
3. The FC can decrease 95.7% and 82.6% strain of the pipeline under compressive and tensile, respectively, compared with soil. Meanwhile, The FC has a better protective effect on the pipeline subjected to compressive than tensile under the fault displacement.

## Data Availability

The datasets generated and analyzed during the current study are available from the corresponding author on reasonable request.
